# Elevated Troponin Serum Levels in Adult Onset Still's Disease

**DOI:** 10.1155/2015/732095

**Published:** 2015-02-12

**Authors:** Carlo Umberto Manzini, Lucio Brugioni, Michele Colaci, Maurizio Tognetti, Amelia Spinella, Marco Sebastiani, Dilia Giuggioli, Clodoveo Ferri

**Affiliations:** ^1^Chair and Rheumatology Unit, Medical School, Azienda Ospedaliero-Universitaria, University of Modena and Reggio Emilia, Policlinico di Modena, Via del Pozzo 71, 41100 Modena, Italy; ^2^Critical Care Unit, Azienda Ospedaliero-Universitaria Policlinico di Modena, Modena, Italy

## Abstract

Adult onset Still's disease (AOSD) is a rare inflammatory systemic disease that occasionally may affect myocardium. Diagnosis is based on typical AOSD symptoms after the exclusion of well-known infectious, neoplastic, or autoimmune/autoinflammatory disorders. In the case of abrupt, recent onset AOSD, it could be particularly difficult to make the differential diagnosis and in particular to early detect the possible heart involvement. This latter event is suggested by the clinical history of the four patients described here, incidentally observed at our emergency room. All cases were referred because of acute illness (high fever, malaise, polyarthralgias, skin rash, and sore throat), successively classified as AOSD, and they presented abnormally high levels of serum troponin without overt symptoms of cardiac involvement. The timely treatment with steroids (3 cases) or ibuprofen (1 case) leads to the remission of clinicoserological manifestations within few weeks. These observations suggest that early myocardial injury might be underestimated or entirely overlooked in patients with AOSD; routine cardiac assessment including troponin evaluation should be mandatory in all patients with suspected AOSD.

## 1. Introduction

Adult onset Still's disease (AOSD) is a rare inflammatory disease characterized by spiking fevers >39°C, arthritis/arthralgias, typical salmon-colored bumpy rash, and possible involvement of visceral organs [1]. The etiology of AOSD remains obscure, but an autoimmune pathogenesis has been suggested [2]. The age peak of disease onset is between 16 and 35 years, with an incidence below 1/100,000 inhabitants, without gender differences. The diagnosis is exclusively based on clinical symptoms, according to the criteria proposed by Yamaguchi et al. [3] or Cush et al. [4], after the exclusion of well-known infectious, neoplastic, or other autoimmune/autoinflammatory disorders [5]. Corticosteroids and nonsteroidal anti-inflammatory drugs represent the first line therapy in the acute phase, while disease-modifying antirheumatic drugs (sulphasalazine, hydroxychloroquine, or methotrexate) or anti-TNF*α* blockers can be considered in the chronic subsets.

Among AOSD clinical manifestations, serositises, including pericardial effusion, are also described (approximately 30–40% of cases), as well as myocardium/endocardium involvement [6–13]. Pericardial complication can be one of the presenting symptoms [8, 14, 15] or manifestation of relapsing disease [16–18]. In this context, increased troponin levels have also been reported in anecdotal observations, probably related to heart involvement [14]. Troponin is a protein found only in cardiac myocytes; it exerts an important role in regulating the interaction of actin and myosin filaments during cardiac contraction. Because of its cardiac origin, troponin is commonly used as a very sensitive and specific marker of myocardial damage, not only in the case of myocardial ischemia, but also in inflammatory diseases inducing cardiac injury, such as AOSD [19].

We describe four consecutive patients, referred to the emergency room of our university-based hospital, who presented abnormally increased levels of serum troponin in the absence of significant electrocardiographic and echocardiographic abnormalities, successively diagnosed as AOSD.

## 2. Case Reports

### 2.1. Case Number 1

A 19-year-old man was referred to the emergency room for sore throat and high fever up to 39°C, skin rash of the trunk and limbs, and polyarthralgias, poorly responsive to acetaminophen (Table 1). Blood exams revealed an increase of C-reactive protein (CRP 21.8 mg/dL; n.v. 0–0.7 mg/dL), erythrocyte sedimentation rate (ESR 74 mm/h, normal values 0–15 mm/h), ferritin (1,503 ng/mL, normal values 13–150 ng/mL), and WBC (19 × 10^3^/*μ*L with neutrophil count 92%). Liver cytolysis and cholestasis indexes were also increased: AST 84 U/L (normal < 31 U/L), ALT 155 U/L (normal < 31 U/L), gamma-GT 242 U/L (normal < 35 U/L), and alkaline phosphatase 483 U/L (reference range 35–129 U/L). Creatine kinase was in normal range. Blood culture, urine culture, and throat swab were negative, as well as virological serology for EBV, CMV, Parvovirus B19, HBV, HCV, and HIV. No abnormalities were observed at chest radiograph and EKG. An increase of troponin cTnI up to 0.5 ng/mL (normal < 0.06 ng/mL) was also noticed. Therefore, transthoracic echocardiography was performed to exclude the presence of possible myocarditis, showing a slight increase of thickness of left ventricular wall with normal systolic function (EF 60%).

According to the Yamaguchi criteria [3], AOSD was diagnosed by a rheumatologist. Intravenous steroid therapy (methylprednisolone) was started, from 60 mg/day (approximately 1 mg/kg body-weight), slowly tapered until the suspension after 40 days. The clinical and laboratory features dramatically improved; in particular, troponin levels were normalized since the third day of therapy. A complete disease remission was observed at subsequent clinical controls.

### 2.2. Case Number 2

A 27-year-old man presented to the emergency room with high fever, polyarthralgias, faint skin rash on the trunk, cough, and dyspnea; previous oral antibiotic therapy prescribed by his family doctor was ineffective (Table 1). Laboratory examinations revealed abnormally increased serum levels of troponin (cTnI up to 3.04 ng/mL), in the absence of chest pain or other signs and/or symptoms suggestive of ischemic heart disease. The EKG was normal, while chest radiograph documented a bilateral parenchymal densification and mild pleural effusion. The echocardiogram and cardiac MRI did not show significant morphofunctional changes, but only a slight pericardial effusion; on the basis of these investigations, ischemic heart disease was excluded.

The patient was hospitalized in the critical care unit; blood pressure was 110/60 mmHg, heart rate 130 bpm (rhythmic), oxygen saturation 96%, and fever 38.8°C. Laboratory examinations showed CRP 14.15 mg/dL 8 (n.v. 0–0.7 mg/dL), WBC 12 × 10^3^/*μ*L (neutrophils 88%), AST 80 U/L and ALT 127 U/L (normal < 31 U/L), and CK 123 (normal < 170 U/L); moreover, antinuclear antibody and rheumatoid factor were negative.

Even if microbiology and virology investigations were negative, broad-spectrum antibiotic intravenous therapy was administered. However, the clinical picture gradually improved only with the use of steroid therapy (methylprednisolone 80 mg/day iv, slowly tapered and discontinued within 38 days); therefore the diagnosis of AOSD was formulated. This treatment leads to stable symptom remission, resolution of lung radiological alteration, and normalization of inflammatory indexes. In particular, serum troponin normalized within the first 4 days of steroid treatment. No flares were observed in the follow-up, up to date.

### 2.3. Case Number 3

A 49-year-old man presented to the emergency room with persistent fever > 40°C for 10 days; he also showed sore throat, polyarthralgias, and faint skin rash on the trunk and neck, not responsive to antibiotic therapy. The physical examination was normal, except for the presence of some painful submandibular lymph nodes (Table 1).

Laboratory examinations revealed leukocytosis (WBC 24 × 10^3^/*μ*L, neutrophils 84.9%), increase of inflammatory indexes (CRP 14.68 mg/dL, ESR 64 mm/h), ferritin (3147 ng/mL), and transaminases (AST/ALT 83/99 U/L); CK was normal. Seriate controls showed slight elevation of troponin cTnI levels up to 0.33 ng/mL (normal < 0.06 ng/mL), in absence of EKG abnormalities. During hospitalization, blood and urine cultures, throat swab, and feces samples, as well as microbiology and virology analysis (Treponema Pallidum, HHV-6, HHV-8, HIV-1, HIV-2, HBV, HCV, Monotest, Parvovirus B19,* B. burgdorferi*,* T. gondii*,* L. pneumophila*, and Quantiferon-TB test) were negative.

The echocardiographic examination permitted excluding endocardial vegetations, while ejection fraction was normal. A total-body computed tomography showed only reactive cervical and abdominal lymph node enlargement.

AOSD diagnosis was performed and steroid therapy at low doses was started (prednisone 25 mg/day, tapered until suspension within the following 3 months); the treatment produced the gradual normalization of inflammatory markers and troponin levels (0.02 after 2 days), with consistent clinical remission.

### 2.4. Case Number 4

A 70-year-old man, with a past clinical history of myocardial infarction in 2008, presented to the emergency room of cardiology unit because of the presence of malaise, fatigue, relapsing high fever, slightly sore throat, and severe arthralgias, during the last 3 months. At the admission, elevated troponin cTnI level (2 ng/mL; Table 1) was found, without new cardiac symptoms or signs suggestive for heart attack. Blood examinations showed leukocytosis (WBC 23 × 10^3^/*μ*L, neutrophils 78.6%) and CRP 18.47 mg/dL. Moreover, antinuclear autoantibodies, rheumatoid factor, and microbiological investigations were negative, as well as EKG, transthoracic echocardiography, and coronary angiography.

The diagnosis of AOSD was formulated. Since the patient refused steroid treatment, ibuprofen 600 mg bid for 6 weeks was prescribed. The symptoms gradually disappeared in 6 weeks, as well as laboratory alterations; in particular, leukocytes, CRP, and troponin levels normalized during the following 7–15 days. No flares were observed in the follow-up, up to date.

## 3. Discussion

The present report described 4 patients with AOSD who presented abnormally increased troponin serum levels at the disease onset; all cases were characterized by monocyclic course of AOSD successfully treated with first line therapy.

AOSD is an inflammatory disorder of unknown etiology, characterized by typical but nonpathognomonic clinical and laboratory features, commonly used to correctly classify the disease after the exclusion of infectious, neoplastic, or autoimmune disorders [20]. To date, even if classification criteria for AOSD have been proposed [1, 2], no specific clinical, serological, and/or instrumental diagnostic markers for AOSD have been identified.

Pericarditis is the most common cardiac manifestation detectable in up to 50% of patients; it is usually subclinical, whereas myocarditis is quite rare [1]. The latter may lead to congestive heart failure and arrhythmias in childhood Still's disease, but these manifestations have rarely been reported in adult patients [12]. In the literature, several cases of AOSD with cardiac manifestation were anecdotally reported. In some patients chest X-ray and cardiac ultrasound showed heart enlargement or pleural effusion; left ventricular hypokinesis and a decreased ejection fraction are also described. Moreover, electrocardiogram could show nonspecific ST-segment and T-wave abnormalities such as T-wave inversion in some leads [14]. More interestingly, Sachs et al. found fibrinoid necrosis at histological examination of myocardial vessels in an AOSD patient [21]. Again, Yamazoe et al. [22] described the case of a 36-year-old female with AOSD complicated by diffuse myocardial hypokinesis at echocardiography and massive pericardial effusion; diagnostic endomyocardial biopsy was performed, revealing fibrosis and infiltration of neutrophils. Acute myocarditis was diagnosed by means of histology for the first time [22]; after pulse intravenous steroids the patient showed marked clinical improvement with complete resolution within one month. Choi et al. [23] reported the case of 37-year-old male with AOSD patient showing T-wave inversions at EKG and increase of troponin serum level, in the absence of significant echocardiographic alterations. Magnetic resonance imaging confirmed the presence of myocarditis, usefully treated with anakinra. Another young man described by Gonzalez et al. [24], who was referred to emergency room for a typical AOSD clinical picture, presented increase of troponin level, ST elevation in leads V1–V5, and diffuse hypokinesia of the left ventricle at echocardiography; remission was achieved by means of high-dosage prednisolone.

The four AOSD patients described here were hospitalized after the initial referring to emergency room, where troponin serum level is routinely measured. This fortuitous chance permitted evidencing the abnormal troponin elevation in all cases; this specific marker alteration prompts us to detect the presence of possible concurrent myocardial damage. The EKG and echocardiography were substantially normal, excluding coronary thrombotic events, while a number of clinical and laboratory features permitted classifying the patients as having AOSD complicated by myocardial inflammatory injury.

Interestingly, early anti-inflammatory treatment induced a rapid remission of the clinical features in all patients, suggesting the presence of possible mild AOSD variants.

Our clinical observations confirmed, at least in part, previous data in the literature, suggesting that myocardial involvement might represent a relatively frequent complication in patients with AOSD. Besides, it is possible to hypothesize that this harmful complication might be underestimated or entirely overlooked in a number of patients, mainly in those with troponin alteration appearing early in the course of AOSD. Therefore, routine cardiac assessment, including troponin evaluation, should be mandatory in all patients with high fever and other inflammatory clinicoserological features evocative of AOSD.

## Figures and Tables

**Table 1:** Clinicoserological features of the 4 patients affected by adult onset Still's disease (AOSD) with serum troponin levels elevation.

Patient number	Sex/age (years)	Clinical features	Troponin cTnI peak normal <0.06 ng/mL	EKG	ECHO-cg	Therapy	Troponin normalization time	AOSD outcome
1	M/19	High fever, rash, sore throat, arthralgias, and neutrophilia	0.5	Normal	Slight LV thickness	Steroids	3 days	Remission
2	M/27	High fever, cough, dyspnea, arthralgias, rash, and neutrophilia	3.04	Normal	Mild pericardial effusion	Steroids	4 days	Remission
3	M/49	High fever, rash, sore throat, arthralgias, and neutrophilia	0.33	Normal	Negative	Steroids	2 days	Remission
4	M/70	High fever, sore throat, arthralgias, and neutrophilia	2.0	Normal	Negative	Ibuprofen	15 days	Remission
